# *Kytococcus schroeteri* Endocarditis

**DOI:** 10.3201/eid1101.040761

**Published:** 2005-01

**Authors:** Cécile Le Brun, Julien Bouet, Philippe Gautier, Jean-Loup Avril, Olivier Gaillot

**Affiliations:** * Centre Hospitalier Universitaire de Rennes, Rennes, France

**Keywords:** letter, heart valve prosthesis, endocarditis, micrococcus, moxifloxacin

**To the Editor:** Becker et al. recently reported the probable implication of *Kytococcus schroeteri* in a case of acute prosthetic valve endocarditis, on the basis of its recovery from blood cultures drawn at the time of infection (*1*). *K. schroeteri* was only characterized on that occasion and is a new micrococcal species resistant to penicillins (*2*). Here, we report the isolation of this organism from prosthetic valve vegetations in a patient who had undergone aortic valve replacement 3 years earlier. The 73-year-old man was admitted with fever (38.8°C) and shortness of breath, which had both increased gradually over the previous 2 months. He had no recent history of intravenous drug administration or catheterization. Laboratory findings showed a leukocyte count of 12 x 10^9^/L (90% neutrophils) and a raised C-reactive protein level. Transesophageal echocardiogram revealed several small vegetations on the Carpentier-Edwards aortic bioprosthesis and a voluminous perivalvular abscess. Four sets of blood cultures were drawn before antimicrobial therapy was inititiated.

Intravenous vancomycin (2 g twice a day) and gentamicin (240 mg/d) were started empirically. The prosthetic material was replaced promptly and the abscess was debrided extensively. Vegetations from the resected material showed numerous polymorphonuclear neutrophils and gram-positive cocci on microscopic examination. Oral rifampicin (600 mg twice a day) was added to the initial regimen.

The postoperative course was uneventful except for cutaneous intolerance to vancomycin, which was replaced with teicoplanin. The physical condition of the patient improved steadily. Gentamicin and rifampicin were discontinued after 3 weeks. Eight months after completion of the 6-week treatment, the patient had no clinical or biologic evidence of infection, although moderate aortic incompetence persisted.

All blood cultures drawn on admission grew gram-positive cocci after 72 hours and subcultures on Trypticase soy agar yielded convex, muddy–yellow colonies of heterogeneous sizes. The vegetations, pus samples of the abscess, and prosthetic valve cultures grew the same type of colonies. All isolates displayed identical biotype and antimicrobial susceptibility and were considered as a single strain. The causative organism (designated ROG140) was initially identified as *Micrococcus* sp. based on the morphologic features, resistance to nitrofurantoin, and inability to grow anaerobically. Assignment to the genus *Kytococcus* was suggested by the arginine dihydrolase activity and resistance to oxacillin, 2 characteristics that are not shared by other micrococci (*3*).

The definitive *K. schroeteri* identification was provided by analysis of the fatty acid content, which was similar to that of the type strain (*2*), and sequencing of the 16S rRNA genes. We sequenced a 1,012-bp fragment encompassing the first two thirds of the 16S rDNA of ROG140 (accession no. AY692224). The sequence was compared with type strains of all members of the former genus *Micrococcus*, and a phylogenetic tree was deduced by the neighbor–joining method (Figure). The sequences of ROG140 and the *K. schroeteri* type strain only differ by an A-to-G substitution at position 747 (*E. coli* numbering). Among the 21 nucleotide differences between the sequences of *K. schroeteri* and the closely related species *K. sedentarius*, 10 are located on a 30-base stretch and constitute a convenient *K. schroeteri* signature (Figure)

**Figure:**
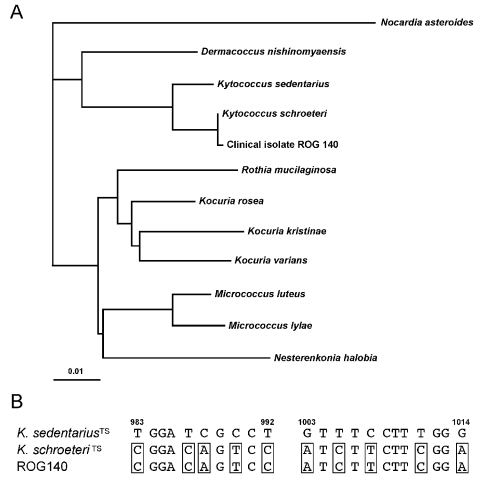
(A) Phylogenetic tree showing relationships among 16S rDNA sequences of clinical isolate ROG140 and type strains of members of the former *Micrococcus* genus. *Nocardia asteroides* was included as an out-group organism. The scale bar represents 1% differences in nucleotide sequences. (B) Sequence alignment of 16S rDNA nucleotides 983-992 and 1003-1014 of *Kytococcus* sp. type strains (^TS^) and clinical isolate ROG140. *K. schroeteri* molecular signatures are boxed. Nucleotide numbering refers to the sequence of the 16S rDNA of *E. coli*.

Antimicrobial susceptibility testing performed with the disk diffusion method and E-tests (AB Biodisk, Solna, Sweden) indicated that the isolate was resistant to penicillins, cephalosporins, kanamycin, tobramycin, erythromycin, clindamycin, sulfonamides, and fusidic acid, but susceptible to imipenem (MIC, 0.25 μg/mL), gentamicin (MIC, 1 μg/mL), trimethoprim (MIC, 0.25 μg/mL), tetracycline (MIC, 0.12 μg/mL), linezolid (MIC, 0.25 μg/mL), vancomycin (MIC, 0.125 μg/mL), teicoplanin (MIC, 0.06 μg/mL), and rifampicin (MIC, <0.002 μg/mL). Unlike the original isolate reported by Becker et al. (*1*), isolate ROG140 was resistant to ofloxacin and ciprofloxacin (MICs, 8 μg/mL). Conversely, moxifloxacin displayed excellent in vitro activity (MIC, 0.05 μg/mL). As moxifloxacin was more rapidly microbicidal than vancomycin in an animal model of *Staphylococcus aureus* prosthetic valve endocarditis (*4*), it might present a potential advantage against infections caused by *K. schroeteri*, especially when the oral route is favored.

The natural habitat of *K. schroeteri* remains unknown. The only isolates of *K.* *schroeteri* identified so far originated from blood or cardiac material, although *Kytococcus* literally means “a coccus from the skin.” Our attempts to recover *K. schroeteri* from the mouth, nose, or skin of our patient were unsuccessful. In a recent study, Szczerba et al. were able to isolate most micrococcal species, including *K. sedentarius* but not *K. schroeteri*, from human skin and mucosa (*5*). However, at that time the authors may not have been aware of this newly described species. The mode of contamination also remains unclear. In the original description (*1*), *K. schroeteri* endocarditis had developed in the patient <3 months after she underwent cardiac surgery, which suggested perioperative contamination. Here, we describe a late onset, subacute infection 3 years after surgery, which is more likely to have been caused by hematogenous spread.

Although *Micrococcus*-like organisms cause endocarditis infrequently (*6*), the description of 2 independent infections due to a new species in a short period is intriguing and suggests a specific pathogenicity, at least on prosthetic heart devices. By demonstrating the presence of the bacteria in the infected site, this report establishes *K.* *schroeteri* as a genuine pathogen in this clinical setting and should prompt further investigations to identify its natural habitat and virulence determinants. At present, commercial systems are not able to identify *K. schroeteri*. However, gram-positive cocci that are strictly aerobic, oxacillin-resistant, and arginine dihydrolase-positive should be recognized as potential *Kytococcus* species and taken into account when endocarditis is suspected.
